# Cross-talk among AFAP1-AS1, ACVR1 and microRNA-384 regulates the stemness of pancreatic cancer cells and tumorigenicity in nude mice

**DOI:** 10.1186/s13046-019-1051-0

**Published:** 2019-02-28

**Authors:** Xu-Bo Wu, Xia Feng, Qi-Meng Chang, Cheng-Wu Zhang, Zhi-Fei Wang, Jie Liu, Zhi-Qiu Hu, Jia-Zhe Liu, Wei-Ding Wu, Zi-Ping Zhang, Xi-Qiang Liu

**Affiliations:** 1 Department of Hepatobiliary and Pancreatic Surgery, Minhang Hospital, Fudan University, No. 170, Xinsong Road, Xinzhuang Town, Minhang District, Shanghai, 201199 People’s Republic of China; 2 Department of Hepatopancreatobiliary Surgery and Minimally Invasive Surgery, Zhejiang Provincial People’s Hospital, People’s Hospital of Hangzhou Medical College, No. 158, Shangtang Road, Zhaohui District, Hangzhou, 310014 Zhejiang Province People’s Republic of China; 3 Key Laboratory of Tumor Molecular Diagnosis and Individualized Medicine of Zhejiang Province, Hangzhou, 310014 People’s Republic of China; 4 Key Laboratory of Gastroenterology of Zhejiang Province, Hangzhou, 310014 People’s Republic of China

**Keywords:** Long non-coding RNA, Actin filament-associated protein 1 antisense RNA 1, MicroRNA-384, Activin receptor a type I, Pancreatic cancer, Cancer stem cell

## Abstract

**Background:**

Pancreatic cancer (PC) represents one of the most aggressive forms of cancer. The role of long non-coding RNAs (lncRNAs) has been highlighted in various malignancies including PC. The aim of the present study was to investigate the effects associated with actin filament-associated protein 1 antisense RNA 1 (AFAP1-AS1) on the progression of PC and the underlying mechanism.

**Methods:**

Microarray-based gene expression profiling of PC was performed to identify PC-related lncRNAs, after which the expression of AFAP1-AS1 and cancer stem cell (CSC) markers in PC tissues and cells were determined accordingly. The potential microRNA-384 (miR-384) capable of binding to AFAP1-AS1, in addition to its ability to regulate activin receptor A type I (ACVR1) were analyzed. In order to investigate the effect of the AFAP1-AS1/miR-384/ACVR1 axis on self-renewal ability, tumorigenicity, invasion, migration and stemness of PC cells, shRNA-AFAP1-AS1, miR-384 mimic and inhibitor were cloned into cells.

**Results:**

High expression of AFAP1-AS1 and ACVR1 with low expression of miR-384 were detected in PC tissues. ACVR1 was determined to be down-regulated when miR-384 was overexpressed, while the inhibition of AFAP1-AS1 decreased its ability to binding competitively to miR-384, resulting in the down-regulation of ACVR1 and enhancing miR-384 expression, ultimately inhibiting the progression of PC. The knockdown of AFAP1-AS1 or overexpression of miR-384 was confirmed to impair PC cell self-renewal ability, tumorigenicity, invasion, migration and stemness.

**Conclusions:**

Taken together, AFAP1-AS1 functions as an endogenous RNA by competitively binding to miR-384 to regulate ACVR1, thus conferring inhibitory effects on PC cell stemness and tumorigenicity.

**Electronic supplementary material:**

The online version of this article (10.1186/s13046-019-1051-0) contains supplementary material, which is available to authorized users.

## Background

Pancreatic cancer (PC) is an aggressive tumor with devastating malignancy capability. The lack of effective early diagnostic and prognostic markers is the largest stumbling block in providing adequate treatment and consequently leads to a poor 5-year survival rate of less than 8% [1]. PC patients are generally diagnosed at a more advanced-stage, with reports suggesting that approximately 50% of patients diagnosed are confirmed to have metastasis [2]. Although existing therapeutic methods such as surgery and radio/chemotherapy are known to aid in lengthening survival and providing symptom relief, relatively few approaches provide a curative effect [3]. Hence, it is of great importance that deeper knowledge pertaining to the underlying molecular mechanisms of PC carcinogenesis and progression are elucidated, in order to identify novel therapeutic and diagnostic targets for cancer treatment.

Long non-coding RNAs (LncRNAs) are involved in a large variety of biological processes, with reports linking the dysregulation of lncRNAs with cancer cell invasion, proliferation and metastasis [4]. LncRNA actin filament-associated protein 1 antisense RNA 1 (AFAP1-AS1) was reported to be up-regulated in nasopharyngeal carcinoma [5], colorectal cancer [6] and cholangiocarcinoma [7]. The up-regulation of AFAP1-AS1 acts as an oncogene and has been demonstrated to result in poor prognoses accompanied by an elevated risk of metastasis [8]. Importantly, pancreatic ductal adenocarcinoma (PDAC) also exhibits high expression of AFAP1-AS1 along with promoted PDAC cell proliferation, invasion and migration [9]. Moreover, accumulating evidence has suggested that cancer stem cells (CSCs) are highly tumorigenic cancer cells with the abilities of self-renewal, differentiation and tumorigenesis [10]. LncRNA metastasis-associated lung adenocarcinoma transcript 1 (MALAT-1) has been linked with the maintenance of pancreatic CSC self-renewal [11]. Moreover, lncRNA have been reported to regulate cancer stem cells by targeting microRNAs (miRNAs) [12]. MicroRNA-384 (miR-384) has been reported to be a target of AFAP1-AS1 based on bioinformatics analysis, while the current study demonstrates that AFAP1-AS1 could decrease miR-384 expression by competitively binding to miR-384 in this study. Additionally, miR-384 has been demonstrated to be down-regulated in renal cell cancer (RCC), with the overexpression of miR-384 indicated to inhibit RCC cell growth and invasion through regulation of its target gene astrocyte elevated gene 1 [13]. Bioinformatics analysis evidence has been provided confirming that Activin A receptor type 1 (ACVR1) is regulated by miR-384, which has been previously shown to play a role in the regulation of stem cell markers [14]. The functional relationship between miR-384 and ACVR1 has been highlighted in breast cancer with studies providing evidence suggesting that miR-384 suppresses breast cancer progression by down-regulating ACVR1 [15]. Based on our exploration of literature, we deem it highly plausible that AFAP1-AS1 could influence the biological characteristics of PC cells via the regulation of ACVR1 by competitively binding to miR-384. Hence, the potential role of AFAP1-AS1 in the initiation and development of PC was investigated in the current study, in order to ascertain as to whether AFAP1-AS1 functions in connection with the AFAP1-AS1/miR-384/ACVR1 pathway to affect PC cell self-renewal ability, tumorigenicity, invasion, migration and stemness.

## Materials and methods

### Ethics statement

All patients enrolled in the study signed informed written consent documents. All experiment protocols were approved by the Clinical Trials and Biomedical Ethics Committee of Zhejiang Provincial People’s Hospital, People’s Hospital of Hangzhou Medical College (2017KT042). The experimental procedure and animal use program of the current study were approved by the Animal Ethics Committee.

### Study subjects

Seventy-five PC tissues and adjacent normal tissues were collected from patients at the Zhejiang Provincial People’s Hospital, People’s Hospital of Hangzhou Medical College between August 2014 and November 2016. There were 39 males and 36 females that were individually diagnostically confirmed post operatively by means of histopathology, among which there were 46 cases ≥50 years old and 29 cases < 50 years old. There were 28 cases with high levels of differentiation, 22 cases of moderate differentiation and 25 cases of poor differentiation. Lymph node metastasis (LNM) was detected in 37 cases, while a total of 38 cases did not exhibit any signs of LNM. Patients with PC were classified in accordance with the tumor-node-metastasis (TNM)-staging-system of the International Union Against Cancer (UICC) [16]. The clinical stages were confirmed to be stage II and stage III. There were 42 stage II cases and 33 cases at stage III. All participating patients did not receive any drug therapy, chemo/radiotherapy or immunobiological treatment prior to inclusion to our study. The human PC cell line SW1990, Capan-1, AsPC-1, MIAPaCa-2, PANC-1 and human normal pancreas cell line HPC-Y5 purchased from Shanghai Yanhui Biological Technology Co., Ltd. (Shanghai, China) were resuscitated. The cell lines were further cultured in Dulbecco’s modified Eagles Medium (DMEM, Gibco Company, Grand Island, NY, USA) containing 10% fetal bovine serum (FBS, Gibco Company, Grand Island, NY, USA) and cultured in an incubator (Thermo Fisher Scientific Inc., Waltham, Massachusetts, USA) with saturated humidity at 5% CO_2_, 37 °C. When the cells were confirmed to have reached a density of 90%, they were treated with 0.25% pulmonary protease (T1300, Beijing Solarbio Science & Technology Co., Ltd., Beijing, China) followed by subculture at a ratio of 1: 3. The human PC cell lines were conducted in connection with reverse transcription quantitative polymerase chain reaction (RT-qPCR) methods for the detection of AFAP1-AS1 expression, with the cell lines screened prior to the subsequent experiments.

### In situ hybridization

PC tissues as well as adjacent normal tissues were sectioned, dewaxed and water saturated. The sections were put on the hybridization oven with 200 μL prehybridization solution added in pre-hybridization at 65 °C for 1 h. During the In situ hybridization, 200 μL AFAP1-AS1 probe hybridization solution (DakoCytomation, Carpinteria, CA, USA) with a final concentration of 500 ng/mL was added. The sections were then cultured in a wet box at 55 °C for 1.5 h, followed by oscillation, washing with 55 °C water for 25 min, and then developed with 2–3 drops of nitro blue tetrazolium (NBT)/5-bromo-4-chloro-3-indolyl phosphate (BCIP) substrate under conditions void of light for 1 h. When staining signal intensity was confirmed to be moderate, pure water was added to terminate the reaction. The sections were then counterstained for nuclei with 200 μL Nuclear Fast Red for about 1–5 min, and rinsed with running water for 10 min. Finally, the sections were dehydrated with 50, 75 and 100% ethanol (5 min/each), sealed with neutral balsam and analyzed for data collection purposes. The chromogenic agent BCIP/NBT was reflected by blue while the counterstaining agent Nuclear Fast Red was red. The staining results were scored in an independent fashion by two pathologists, and the cytoplasm stained with blue was considered to represent the positive cells [17]. Five fields were randomly selected for each section, observed under a microscope (× 200) after which the percentage of positive cells was calculated. The percentage of positive cells < 5% was regarded as negative (−), while ≥5% as positive (+).

### RT-qPCR

Total RNA was extracted using Trizol RNA extraction solution (Invitrogen Inc., Carlsbad, CA, USA). Reverse transcription was performed using Primescript TMRTreagent Kit (RRO37A, Takara Biotechnology Ltd., Dalian, Liaoning, China), according to the following steps: RNA precipitation was dissolved with 40 μL RNAase-free water, followed by adding 12 μL RNAase-free water, 2 μL OdT and 3 μL RNA sample in 200 μL RNAase-free pitot tube, well-mixed. After boiling at 70 °C for 5 min, the tubes were cooled on ice in a prompt manner for 2 min. Then, 1 μL deoxy-ribonucleoside triphosphate (dNTP), 1 μL guanidine isothiocyanate, 5 μL 5 × reverse transcription buffer and 1 μL Moloney Murine Leukemia Virus (MMLV) reverse transcriptase were applied and gently mixed using a pipette, and then further treated with a water bath at 37 °C for 90 min. The reaction was terminated by heating means at 70 °C for 5 min and conserved on an ice box. The target genes as well as internal reference genes were amplified by a fluorescence quantitative PCR instrument (ABI 7500, Applied Biosystems, Carlsbad, CA, USA). A total of 25 μL PCR reaction system was as follows:10 × PCR Buffer, 2.5 μL 25 mmol/L MgCl2, 1.5 μL 10 mmol/L dNTP, 0.5 μL 10 mmol/L Primer, 1 μL 1 nmol/L Probe, 0.25 μL Taq, 2.5 μL cDNA and 15 μL sterile distilled water. The reaction conditions included the following: pre-denaturation at 94 °C for 5 min, 40 cycles of denaturation at 94 °C for 30 s, denaturation at 58 °C for 45 s, denaturation at 72 °C for 30 s, and then extension at 72 °C for 10 min. All the reactions were preset with three replicates. U6 was regarded as the internal reference for AFAP1-AS1 and miR-384, while glyceraldehyde-3-phosphate dehydrogenase (GAPDH) was the internal reference for the other genes. The AFAP1-AS1 and miR-384 expression levels as well as mRNA expression levels of ACVR1, Oct4, Nestin, CK19, CD133, and ABCG2 was calculated using the 2^-ΔΔCt^ method [18], which reflected the ratio of the target gene expression between the experimental group and the control group. The formula applied was as follows: ΔCt = Ct _target genes_ - Ct _GADPH_, ΔΔCT = ΔCt _experimental group_ - ΔCt _control group_. Ct represented the amplification cycles when the real-time fluorescence intensity reached the set threshold value and when the amplification had entered a period of logarithmic growth. The experiment was repeated 3 times. The aforementioned methods were also applicable for the cell experiment. The primer sequences are displayed in Table 1.

**Table 1:** Primer sequences for RT-qPCR

Gene	Sequences
AFAP1-AS1	F: 5′-ACTGAAGAGGAACCAGGGACAG-3′
	R: 5′-GGGGAAACTGAAATGAATGAAG-3′
miR-384	F: 5′-TGTTAAATCAGGAATTTTAA-3′
	R: 5′-TGTTACAGGCATTATGA-3′
ACVR1	F: 5′-GTGAAGGTCTCTCCTGCGGTA − 3′
	R: 5′-GCCATCGTTGATGCTCAGTGA − 3′
Oct4	F: 5’-CAAAGCAGAAACCCTCGTGC-3’
	R: 5’-AACCACACTCGGACCACTCG-3’
Nestin	F: 5’-ATCCCGTCAGCTGGAAAAGG-3’
	R: 5’-GGTGAGCTTGGGCACAAAAG-3’
CK19	F: 5’-ACCATTGAGAACGCCAGGATT-3’
	R: 5’-TCCAGCACCCAAACACTCAA-3’
CD133	F: 5′-TATAAAGCTTACCATGGCCCTCGTACTCGGCTC -3′
	R:5′-TATAGGATCCTCAATGTTGTGATGGGCTTGTC-3′
ABCG2	F: 5′-CAGGTGGAGGCAAATCTTCGT-3′
	R: 5′-CTTGTACTCCGTCAGCGTGA-3′
GAPDH	F: 5’-ACAGTCCATGCCATCACTG-3’
	R: 5’-AGTAGAGGCAGGGATGATG-3’
U6	F: 5’-TGCGGGTGCTCGCTTCGGCAGC-3′
	R: 5′-CCAGTGCAGGGTCCGAGGT-3′

Note: RT-qPCR, reverse transcription quantitative polymerase chain reaction; AFAP1-AS1, actin filament-associated protein 1 antisense RNA 1; miR-384, microRNA-384; ACVR1, activin receptor A type I; Oct4, organic cation/carnitine transporter 4; CK19, keratin 19; ABCG2, breast cancer resistance protein; GAPDH, glyceraldehyde-3-phosphate dehydrogenase; F, forward; R, reverse

### Western blot analysis

The total protein of the cells was extracted using a radioimmunoprecipitation assay (RIPA) lysate (R0010, Beijing Solarbio Science & Technology Co., Ltd., Beijing, China) containing phenylmethylsulfonyl fluoride (PMFS). The cells were then incubated on ice for 30 min, centrifuged at 28985×g (4 °C) for 10 min followed by collection of the supernatant. The concentration of each protein sample was subsequently determined using bicinchoninic acid (BCA) kit (23,225, Pierce, Rockford, IL, USA), and adjusted with deionized water. Preparation of 10% SDS-PAGE gel was prepared (P0012A, Beyotime Biotechnology Co., Ltd., Shanghai, China). In the next step, each well was added with 50 μg protein sample, and the electrophoresis was at a stable pressure of 80 (Volt) V for 2 h. The protein was transferred onto polyvinylidene fluoride film (PVDF, Millipore, Billerica, MA, USA) via the wet transfer method at 110 V for 2 h. Membrane blockade was then performed with Tris-buffered saline with Tween 20 (TBST) containing 5% skimmed milk powder for 2 h. After the blocking solution was discarded, the film was washed once with TBST and incubated overnight at 4 °C with the primary rabbit antibodies against ACVR1 (1: 1000, ab155981), Oct4 (1: 1000, ab19857), Nestin (1: 2000, ab7659), CK19 (1: 1000, ab15463), CD133 (1: 500, ab16518), ABCG2 (1: 500, ab24115), and GAPDH (1: 2500, ab9485). All the above antibodies were purchased from Abcam Inc. (Cambridge, MA, USA). The film was then washed 3 times with TBST (10 min/time), and washed an additional 3 times with poly (butylene succinate-co-terephthalate) (PBST, 10 min/time). Finally, the film was developed with electrogenerated chemiluminescence (ECL) solution (WBKLS0100, Millipore, Billerica, MA, USA), with images then developed after exposure in a dark box. The relative protein expression was the ratio of target band to the internal reference GAPDH band. ImageJ (Bio-Rad Inc., Hercules, CA, USA) software was employed in the analysis of the grey value of protein bands. The experiment was repeated three times.

### Flow cytometry

The culture medium was discarded when cell growth had reached the logarithmic phase. The cells were washed once with phosphate buffer saline (PBS), and further detached with ethylene diamine tetraacetic acid (EDTA)-free trypsin in an incubator at 37 °C for 2 min. When the cells were observed to have become round in shape, the digestion process was terminated by adding approximately 5 mL culture medium containing serum. Next, the cells were well mixed and placed into a sterilized centrifuge tube and centrifuged at 453×g for 5 min with the supernatant removed. The cells were then washed with PBS, suspended with 500 μL PBS, with CSC sorting performed on a flow cytometer. Finally, the sorted CSCs were maintained in DMEM/F-12 complete medium (containing 1% penicillin, streptomycin and B27) containing 10% FBS and cultured in a CO_2_ incubator at 37 °C.

### Cell grouping and transfection

The PC cells PANC-1 and SW1990 at the logarithmic growth phase subsequently underwent transfection. The cells were assigned into shRNA-negative control (NC) group (PC cells transfected with shRNA-NC vector), shRNA-AFAP1-AS1–1 group (PC cells transfected with shRNA-AFAP1-AS1–1 vector), shRNA-AFAP1-AS1–2 group (PC cells transfected with shRNA-AFAP1-AS1–2 vector), empty vector group (PC cells transfected with empty vector), AFAP1-AS1 group (PC cells transfected with AFAP1-AS1 overexpression vector), miR-384 mimic-NC group (PC cells transfected with miR-384 mimic NC), miR-384 mimic group (PC cells transfected with miR-384 mimic), miR-384 inhibitor-NC group (PC cells transfected with miR-384 inhibitor NC), miR-384 inhibitor group (PC cells transfected with miR-384 inhibitor) and miR-384 inhibitor + shRNA-AFAP1-AS1–1 group (PC cells transfected with miR-384 inhibitor + shRNA-AFAP1-AS1–1 vector). All the transfection vectors or plasmids were synthetized by Shanghai Sangon Biotechnology Co. Ltd. (Shanghai, China).

The PC cells were passaged one day prior to transfection, and seeded into 6-well plates (1 × 10^5^ cells/well). Cell transfection was performed when cell confluence reached 70–80% and in accordance with the instructions of Lipofectamine® 2000 reagent (11,668,019, Invitrogen Inc., Carlsbad, CA, USA). A total of 250 μL serum-free DMEM was used to dilute 100 pmoL shRNA-AFAP1-AS1–1, shRNA-AFAP1-AS1–2, shRNA-NC, miR-384 mimic-NC, miR-384 mimic, miR-384 inhibitor-NC, miR-384 inhibitor and miR-384 inhibitor + shRNA-AFAP1-AS1–1 (a final concentration of 50 nM), mixed in a gentle manner and incubated at room temperature for 5 min. An additional 250 μL serum-free DMEM was used to dilute 5 μL of Lipofectamine 2000, gently mixed and incubated at room temperature for 5 min. Next, the two aforementioned properties were mixed well, incubated at room temperature for 20 min and then added to the cell culture wells. The transfected cells were continually cultured in a 5% CO_2_ incubator at 37 °C for 6–8 h. After the introduction of a complete culture medium, a further 24–48 h period of culturing was performed to further process the experiment.

### Sphere formation assay

The cells at the logarithmic growth phase were constructed into a single cell suspension, tallied using a hemocytometer and diluted to 10^3^ orders of magnitude for use by 10 × gradient dilution method. A total of 3 mL of cell culture medium containing 0.1% agar was prepared in the 15 mL centrifuge tube, mixed on the whirlpool mixer and then added to 300 cells, further mixing on a whirlpool mixer was then performed. One mL of liquid (containing 100 cells) for each group was inoculated into the 6-well plates, and shaken in a prompt fashion for even distribution purposes. Three duplicates were set for each well. When the agar was confirmed to have taken shaped, 1 mL fresh medium was added on superior surface and incubated in a 5% CO_2_ saturated incubator at 37 °C for 7–10 days. The number of spheres was counted under a microscope.

### Colony formation assay

The cells at the logarithmic growth phase were made into the single cell suspension, counted with a hemocytometer and diluted to 100 μL medium containing 1 cell by using 10 × gradient dilution method. The two kinds of cells were inoculated into the 96-well plates respectively, followed by the addition of 100 μL diluted cell suspension. The number of successfully inoculated wells was counted under a microscope, and then incubated in a 5% CO_2_ saturated incubator at 37 °C for 7–10 days. The number of wells with clone spheres (> 50 cells) was counted under a microscope, and the colony formation rate was calculated as follows: (number of wells with clone spheres/number of successfully inoculated wells) × 100%.

### 5-ethynyl-2′-deoxyuridine (EdU) staining

The cells at the logarithmic growth phase were seeded into the 96-well plates with 6000–10,000 cells per well and incubated in an incubator overnight. An EdU kit (C00031Apollo®567, RiboBio Co., Ltd., Guangzhou, Guangdong, China) was applied to detect cell proliferation. The cells were labeled by EdU: each well was added with 100 μL medium containing 50 μmol/L EdU solution and incubated for 2 h. After the cells had been fixed with 4% paraformaldehyde for 30 min and cleared with 0.5% TritonX-100 for 10 min, nuclear staining was performed with the addition of 100 μL Apollo® staining solution under conditions void of light. All the nuclei were stained blue (Hoechst staining) under a microscope, while the nuclei of the proliferated cells were stained red.

### Transwell assay

A total of 50 mg/L of Matrigel (Sigma-Aldrich, SF, CA, USA) was diluted at a ratio of 1: 8. The membrane at the apical chamber was coated with 60 μL diluted Matrigel, and air-dried at room temperature. After the residual liquid in the culture plate had been aspirated, 50 μL of serum-free culture medium containing 10 g/L bovine serum albumin (BSA) was added to each well and placed at 37 °C for 30 min. Cells at the logarithmic growth phase were obtained from each group, and the cell density was adjusted to 1 × 10^5^ cells/mL with serum-free culture medium containing 10 g/L BSA. Next, 200 μL cell suspension was added to the Transwell chamber, while the basolateral chamber of the 24-well plates was added with 500 μL culture medium containing 100 mL/L FBS. The Transwell chamber was placed in a culture plate and incubated in a 5% CO_2_ incubator at 37 °C for 24 h. After a 24-h period of incubation, the cells on the inner side of the chamber closer in proximity to the PVPF membrane were removed using a cotton swab, fixed with 95% alcohol for 30 min at room temperature, stained with crystal violet (Sigma-Aldrich, SF, CA, USA) for 20 min and then rinsed 3 times with water. Finally, the number of invasive cells was counted under the guidance of an inverted microscope (CKX41SF, Olympus, Tokyo, Japan).

### Fluorescence in situ hybridization (FISH) assay

The subcellular localization of AFAP1-AS1 in cells was identified using a FISH assay. In accordance with the instructions of Ribo™ lncRNA FISH Probe Mix (Red) (RiboBio Co., Ltd., Guangzhou, Guangdong, China), the specific method applied was performed as follows: the coverslips were placed in the 6-well plates, with the cells at the logarithmic growth phase inoculated with the cells/well. When cell confluence had reached approximately 80% after 1 day culture, the coverslips were fixed in 4% paraformaldehyde at room temperature after PBS washing. The cells were subsequently treated with 2 μg/mL protease K, glycine and phthalide reagent, added with 250 μL prehybridization solution and incubated at 42 °C for 1 h. After that, 250 μL hybridization solution containing 300 ng/mL probe was added after the prehybridization solution removed, and further incubated at 42 °C overnight. The next day, the cells were washed 3 times with PBST, after which 4′,6-diamidino-2-phenylindole (DAPI) staining solution diluted with PBST was added at a ratio of 1: 800 to the 24-well plate for nucleus staining over a period of 5 min. After three PBST washes (3 min/time), the coverslips were sealed with an anti-fluorescence quencher. Finally, 5 different fields were selected under the guidance of a fluorescence microscope (Olympus, Tokyo, Japan), observed and photographed accordingly.

### RNA pull down

Magnetic RNA-Protein Pull-Down kit (Pierce, Rockford, IL, USA) was applied, 1 μg biotin-labeled RNA AFAP1-AS1 was placed into Eppendorf (EP) tubes, added with 500 μL Structure Buffer, water bathed at 95 °C for 2 min, followed by ice bathing for 3 min. A total of 50 μL fully resuspended beads were incubated in EP tubes at 4 °C overnight. The beads were subsequently centrifuged at 1812×g for 3 min with the supernatant discarded, followed by three 500 μL RNA binding protein immunoprecipitation (RIP) wash buffer rinses. Then, 10 μL cell lysate was added and placed at room temperature for 1 h. The incubated bead-RNA-protein mixture was centrifuged at a low speed with the supernatant recycled, and washed 3 times with 500 μL RIP wash buffer. Next, 10 μL cell lysate supernatant was used as protein Input. After the protein concentration had been determined, western blot analysis methods were performed in order to measure protein expression. The experiment was repeated 3 times.

### RIP assay

The AFAP1-AS1 binding ability to Argonaute 2 (AGO2) protein was detected using the RIP kit (Millipore, Bedford, MA, USA). The cells were washed with pre-cooled PBS, after which the supernatant was discarded. The cells were then lysed using an equal volume of radioimmunoprecipitation (RIPA) lysate (P0013B, Beyotime Biotechnology Co., Shanghai, China) in an ice bath for 5 min, centrifuged (4 °C) at 39452×g for 10 min, with the supernatant was extracted. A portion of the cell extract was used as an Input, and the other portion was incubated with antibodies for co-precipitation. Each co-precipitation reaction system was washed with 50 μL of beads and resuspended in 100 μL of RIP wash buffer. Each group was added with 5 μg antibodies for combination purposes. The magnetic bead-antibody complex was then washed and resuspended in 900 μL RIP wash buffer, followed by incubation with 100 μL cell extract at 4 °C overnight. The samples were then placed on a magnetic base to collect the magnetic bead-protein complexes. The samples and Inputs were then digested with proteinase K for RNA extraction, which was then used for western blot analysis. The antibodies used for RIP assay were AGO2 (ab32381, 1: 2000, Abcam Inc., Cambridge, MA, USA) diluted at room temperature for 30 min, and Immunoglobulin G (IgG) (ab172730, 1: 100, Abcam Inc., Cambridge, MA, USA) used as the NC. The experiment was repeated 3 times.

### Dual-luciferase reporter gene assay

The target gene of AFAP1-AS1 and its targeting relationship to miR-384 were analyzed and predicted using the online prediction website RNA22. The AFAP1-AS1–3’UTR gene fragment was synthesized, and cloned into pmirGLO (Promega, Madison, WI, USA) via the endonuclease sites Spe I and Hind III. The mutant (Mut) sites of complementary sequence on the AFAP1-AS1 wide type (DCLK1-Wt) plasmid were designed. After restriction endonuclease digestion, the target fragment was inserted into the pMIR-reporter plasmids by using T4 DNA ligase. The luciferase PRL-TK vector expressed renilla (TaKaRa, Dalian, Liaoning, China) was used as the internal control in order to adjust the difference between the number of cells and the transfection efficiency. The miR-384 mimic as well as the NC were co-transfected in a respective manner with a luciferase reporter gene vector into PANC-1 cells for 48 h, after which the cells were collected and lysed. Finally, the luciferase activity was assessed using the Dual-Luciferase Reporter Assay System (Promega, Madison, WI, USA). The experiment was repeated 3 times.

The online prediction website microRNA.org was applied to predict and analyze the target gene of miR-384 and its targeting relationship between ACVR1. ACVR1–3’UTR gene fragment was synthesized, and cloned into pmirGLO (Promega, Madison, WI, USA) via the endonuclease sites Spe I and Hind III. The Mut sites of complementary sequence on an ACVR1-Wt plasmid were designed. After restriction endonuclease digestion, the target fragment was inserted into the pMIR-reporter plasmids using T4 DNA ligase. The luciferase PRL-TK vector expressing renilla (TaKaRa, Dalian, Liaoning, China) was used as the internal control and utilized when adjusting for the difference between the number of cells and the efficiency of transfection. The miR-384 mimic and NC were respectively co-transfected with luciferase reporter gene vector into PANC-1 cells for 48 h, after which the cells were collected and lysed accordingly. Finally, luciferase activity was examined using the Dual-Luciferase Reporter Assay System (Promega, Madison, WI, USA). The experiment was repeated 3 times.

### Tumor formation in nude mice

Twenty-four specific-pathogen-free (SPF) male nude mice (4-week-old, 18–20 g) were purchased from Shanghai SLAC laboratory animal Co., Ltd. (Shanghai Laboratory Animal Center of Chinese Academy of Sciences, Shanghai, China). The mice were housed under stable temperature (25 °C–27 °C) conditions at a stable humidity of 45–50%. The mice were randomly assigned into 4 groups (shRNA-NC, shRNA-AFAP1-AS1–1, miR-384 inhibitor-NC and miR-384 inhibitor groups) with 6 mice placed in each group and inoculated with transfected cells after anesthesia administration. In brief, the cells exhibiting logarithmic growth following transfection were resuspended in 50% Matrigel (BD Biosciences, Bedford, MA, USA) with cell density adjusted 1 × 10^7^ cells/mL. A single cell suspension of 0.5 mL containing 5 × 10^6^ of transfected cells was subcutaneously injected into each mouse at the left under-axillary. On the 1st, 2nd, 3rd, 4th, 5th week, the tumor size of nude mice was measured with using a Vernier caliper. Tumor volume (mm^3^) = (L × W^2^)/2 (L stood for the tumor length and W stood for tumor width). The experiment was repeated 3 times.

### Statistical analysis

All data were analyzed by SPSS 21.0 software (IBM, Armonk, NY, USA). Measurement data were expressed as mean ± standard deviation. Paired *t*-test was applied for comparison between tumor tissues and adjacent normal tissues, while unpaired *t*-test was used for comparisons between the other two groups. Comparisons among multiple groups were analyzed by analysis of variance (ANOVA). In the event that variance was equal, Q test was applied for pairwise comparison. When the variance was uneven, a non-parametric rank test was used. Repeated measures ANOVA was used to analyze the data at different time points during the experiment. The measurement data were expressed as a percentage (%), and the data were analyzed by the chi-square test. A value of *p* < 0.05 was considered to be statistically significant.

## Results

### AFAP1-AS1 is involved in PC progression

**Fig. 1:**
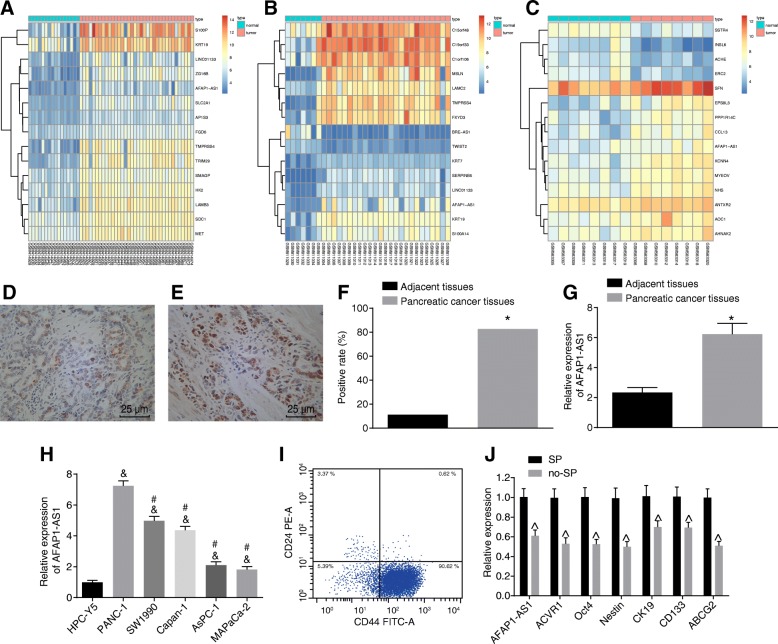
Involvement of AFAP1-AS1 in the development and progression of PC. **a**, **b** and **c**, the thermal map of PC-related GSE16515, GSE32676 and GSE22780 chips, respectively; **d** and **e**, the positive expression of AFAP1-AS1 in cancer tissues and adjacent normal tissues measured by In situ hybridization; **f**, statistical chart of AFAP1-AS1 positive expression; **g** and **h**, RT-qPCR analysis of AFAP1-AS1 expression in PC tissues and cell lines; **i**, the SP cells with CSC-like properties sorted by flow cytometry; **j**, RT-qPCR analysis of the expression of CSC markers Oct4, ABCG2, Nestin, CK19 and CD133 as well as AFAP1-AS1 and ACVR1. ^*^, *p* < 0.05 vs. the PC tissues (*n* = 75); ^&^, *p* < 0.05 vs. the HPC-Y5 cell line; ^#^, *p* < 0.05 vs. the PANC-1 cell line; ^^^, *p* < 0.05 vs. the SP cells. The measurement data were expressed as mean ± standard derivation; paired *t*-test (in panel G) or *t*-test (in panel **j**) was performed for comparison between two groups; one-way ANOVA was applied for comparison among multiple groups. The data in panel **f** were enumeration data, and analyzed by Chi-square test. The experiment was repeated three times. AFAP1-AS1, actin filament-associated protein 1 antisense RNA 1; PC, pancreatic cancer; RT-qPCR, reverse transcription quantitative polymerase chain reaction; SP, side population; CSC, cancer stem cell; ACVR1, activin receptor A type I; ABCG2, ATP-binding cassette subfamily G member 2

Data were initially screened from the GEO database. As illustrated in Fig. 1a-c, the expression of AFAP1-AS1 was up-regulated in GSE16515, GSE32676 and GSE22780. A FISH assay was subsequently conducted in order to determine AFAP1-AS1 localization. The results obtained indicated that AFAP1-AS1 was predominately localized in the cytoplasm with a diffuse or granular distribution. A minor amount of cells exhibited AFAP1-AS1 expression in both nucleus and cytoplasm. Certain tissues also exhibited cytoplasmic staining in a few interstitial cells (Fig. 1d-e). Compared with cancer tissues (82.67%, 62/75), the positive expression rate of AFAP1-AS1 in the adjacent normal tissues (11.11%, 8/75) was markedly reduced (*p* < 0.05) (Fig. 1f). The correlation analysis between the AFAP1-AS1 expression and the characteristics of PC patients (Table 2) demonstrated that the expression of AFAP1-AS1 was not correlated with the gender, age, local infiltration and differentiation degree (*p* > 0.05), however a positive correlation to TNM stage, lymph node metastasis, and tumor size was observed (*p* < 0.05), suggesting that AFAP1-AS1 expression is positively correlated with the PC progression. The RT-qPCR results indicated that the expression of AFAP1-AS1 was significantly increased in cancer tissues in contrast to that of the adjacent normal tissues (*p* < 0.05) (Fig. 1g). Additionally, the expression of AFAP1-AS1 in the PC cell line was significantly higher than that of the HPC-Y5 cell line (*p* < 0.05), while the expression of AFAP1-AS1 in SW1990, Capan-1, AsPC-1 and MIAPaCa-2 cell lines were markedly decreased in comparison with the PANC-1 cell line (*p* < 0.05). Thus, the PANC-1 cell line was employed for further experiments. Flow cytometry analysis revealed the ratio of cancer side population (SP) cells with CSC-like properties was 0.62% (Fig. 1i), while the RT-qPCR, results as depicted in Fig. 1j demonstrated that the expression of AFAP1-AS1 and ACVR1 was significantly decreased while the expression of the CSC markers Oct4, ABCG2, Nestin, CK19 and CD133 in non-SP cells compared with that in SP cells was diminished (*p* < 0.05). The CD24^+^ and CD44^+^ stem cells were noted to be isolated from SW1990 and Capan-1, with the results obtained indicating that the expression of AFAP1-AS1 and CSC markers was higher in the SP cells when compared to the non-SP cells (Additional file 1: Figure S1), which highlighted the potential universal role of AFAP1-AS1 in pancreatic CSCs. The aforementioned results suggested that AFAP1-AS1 might participate in the development and progression of PC.

**Table 2:** Correlation between AFAP1-AS1 expression and clinical characteristics in patients with PC

Characteristics	Cases	AFAP1-AS1		
		Positive cases (%)	χ^2^	*p*
Gender			0.592	0.442
Male	39	34 (87.18)		
Female	36	28 (77.78)		
Age (years)			0.852	0.356
< 50	29	22 (75.86)		
≥ 50	46	40 (86.96)		
TMN stage			3.916	0.048
II	42	31 (73.81)		
III	33	31 (93.94)		
Lymph node metastasis			5.701	0.017
Yes	37	35 (94.59)		
No	38	27 (71.05)		
Tumor size (cm)			5.396	0.020
< 5 cm	33	23 (69.70)		
≥ 5 cm	42	39 (92.86)		
Differentiation degree			0.812	0.368
Poor	25	22 (88.00)		
Moderate	22	18 (81.82)		
High	28	22 (78.57)		
Local infiltration			0.729	0.393
Yes	34	30 (88.24)		
No	41	32 (78.05)		

Note: AFAP1-AS1, actin filament-associated protein 1 antisense RNA 1; PC, pancreatic cancer; TNM, tumor-node-metastasis. *N* = 75

### Knockdown of AFAP1-AS1 inhibits the PC cell stemness

**Fig. 2:**
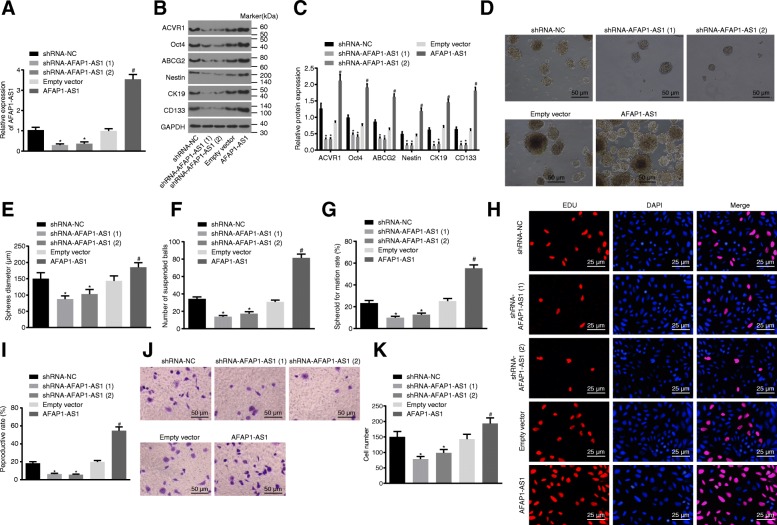
Effect of inhibition of AFAP1-AS1 on PANC-1 cell stemness. **a**, RT-qPCR analysis of AFAP1-AS1expression; **b**, western blot analysis of CSC makers; **c**, statistical chart of protein expression; **d**, sphere formation of PANC-1 cells (× 200); **e** and **f**, statistical chart of sphere diameter and spheres per 100 cells of PANC-1 cells determined by sphere formation assay; **g**, statistical chart of monoclonal formation rate evaluated by clone formation assay; **h**, proliferation of PANC-1 cells tested by EdU staining (× 200); **i**, statistical chart of cell proliferation; **j**, the number of invasive cells determined by Transwell assay (× 200); **k**, statistical chart of cell invasion; ^*^, *p* < 0.05 vs. the shRNA-NC group. #, *p* < 0.05 vs. the empty vector group. All the above data was measurement data and expressed as mean ± standard derivation. One-way ANOVA was applied for comparison among three groups. The *t*-test was conducted for the comparison between two groups. The experiment was repeated three times. AFAP1-AS1, actin filament-associated protein 1 antisense RNA 1; PC, pancreatic cancer; RT-qPCR, reverse transcription quantitative polymerase chain reaction; GAPDH, glyceraldehyde-3-phosphate dehydrogenase; ACVR1, activin receptor A type I; ABCG2, ATP-binding cassette subfamily G member 2

Initially, shRNA-AFAP1-AS1–1 and shRNA-NC sequences, as well as AFAP1-AS1 overexpression and empty vectors were transfected into PANC-1 and SW1990 cells, in an attempt to assess the interfering effect of shRNA on the expression of AFAP1-AS1. The RT-qPCR results (Fig. 2a, Additional file 2: Figure S2A), indicated that the expression of AFAP1-AS1 in both shRNA-AFAP1-AS1–1 and shRNA-AFAP1-AS1–2 groups was notably decreased when compared with that in the shRNA-NC group (*p* < 0.05); while in comparison to the empty vector group, revealed that the expression of AFAP1-AS1 was elevated in the AFAP1-AS1 group (*p* < 0.05), suggesting that AFAP1-AS1 expression was interfered with by shRNA targeting AFAP1-AS1 and overexpressed by AFAP1-AS1 overexpression vector. Western blot analysis revealed that compared with the shRNA-NC group, the expression of CSC markers Oct4, ABCG2, Nestin, CK19 and CD133 as well as ACVR1 were decreased in the shRNA-AFAP1-AS1–1 and shRNA-AFAP1-AS1–2 groups while the AFAP1-AS1 group displayed increased expression of the above CSC markers (*p* < 0.05) (Fig. 2b-c, Additional file 2: Figure S2 BC). Then, sphere formation and clone formation assays were performed, exhibiting fewer cell spheres, smaller microsphere diameter and lower monoclonal rate in the shRNA-AFAP1-AS1–1 and shRNA-AFAP1-AS1–2 groups than that in the shRNA-NC group, while the AFAP1-AS1 group showed opposite trend (Fig. 2d-g, Additional file 2: Figure S2D-G). Finally, EdU staining and Transwell assay revealed that the number of proliferative and migrated cells had diminished in the shRNA-AFAP1-AS1–1 and shRNA-AFAP1-AS1–2 groups compared with that in the control group, while the AFAP1-AS1 group showed opposite trend (*p* < 0.05) (Fig. 2i-k). Taken together, our findings demonstrated that AFAP1-AS1 could maintain stemness of pancreatic CSCs, while inhibition of AFAP1-AS1 exerted suppressive effects on sphere formation, proliferation, invasion and stemness of PC cells.

### AFAP1-AS1 regulates ACVR1 through competitively binding to miR-384

**Fig. 3:**
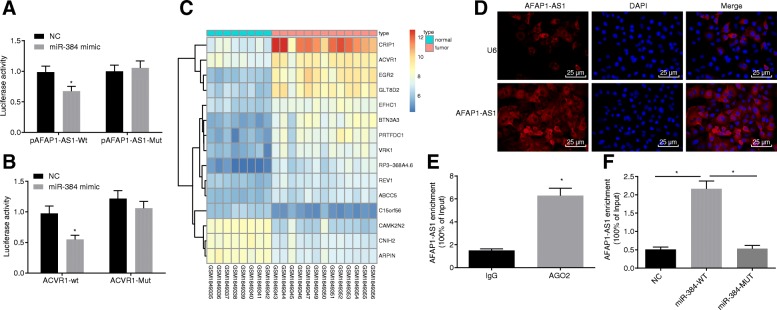
AFAP1-AS1 is involved in regulation of ACVR1 through competitively binding to miR-384. **a**, AFAP1-AS1 targeted to inhibit miR-384 as verified by dual-luciferase reporter gene assay; ^*^, *p* < 0.05 vs. the NC group; **b**, miR-384 targeted to inhibit ACVR1 as confirmed by dual-luciferase reporter gene assay; ^*^, *p* < 0.05 vs. the NC group; **c**, heat map of PC-related GSE71989 chip; **d**, localization of AFAP1-AS1 in cells detected by FISH; **e**, the binding ability of AFAP1-AS1 to AGO2 evaluated by RIP assay; ^*^, *p* < 0.05 vs. the IgG group. **f**, the binding ability of AFAP1-AS1 to miR-384 determined by RNA pull down. All the above data was measurement data and expressed as mean ± standard derivation. The *t*-test was performed for comparison between two groups, and one-way ANOVA was applied for comparison among multiple groups. The experiment was repeated three times. AFAP1-AS1, actin filament-associated protein 1 antisense RNA 1; NC, negative control; PC, pancreatic cancer; ACVR1, activin receptor A type I; miR-384, microRNA-384; FISH, fluorescence in situ hybridization; RIP, RNA binding protein immunoprecipitation; AGO2, Argonaute 2

The targeting relationship between AFAP1-AS1 and miR-384 was predicted by bioinformatics prediction website RNA22, and further confirmed through the application of a dual-luciferase reporter gene assay. The results of the bioinformatics analysis suggested that AFAP1-AS1 could target and inhibit miR-384. As illustrated in Fig. 3a, when compared with the NC group, the fluorescence intensity of pAFAP1-AS1-Wt was notably decreased in the miR-384 mimic group (*p* < 0.05), while the fluorescence intensity of pAFAP1-AS1-Mut did not display a distinct difference between the NC and miR-384 mimic groups (*p* > 0.05). The results suggested that AFAP1-AS1 could competitively combine to miR-384. Meanwhile, the targeting relationship between miR-384 and ACVR1 was investigated in connection with the online database (microRNA.org). The results obtained indicated that ACVR1 was negatively regulated by miR-384. A dual-luciferase reporter gene analysis demonstrated that the fluorescence intensity of pACVR1-Wt was markedly decreased in the miR-384 mimic group compared with NC group (*p* < 0.05), while no significant difference in regard to the fluorescence intensity of pACVR1-Mut was detected between the NC and miR-384 mimic groups (*p* > 0.05) (Fig. 3b). The data analysis of GSE71989 chip suggested that the expression of ACVR1 was significantly higher in the PC than in the pancreatic tissues (Fig. 3c). The localization of AFAP1-AS1 in cells was detected by FISH, the results of which demonstrated that AFAP1-AS1 was primarily localized in the cytoplasm (Fig. 3d). Further RIP in connection with RNA pull-down assays were performed to determine the binding ability of AFAP1-AS1 to AGO2 and AFAP1-AS1 to miR-384, respectively. In contrast to IgG, AFAP1-AS1 enrichment increased in AGO2, suggesting that AFAP1-AS1 could bind to AGO2 (Fig. 3e). Furthermore, AFAP1-AS1 enrichment increased in Mut-miR-384 compared with mut-miR 384 (Fig. 3f). Our results provided evidence regarding the mechanism that AFAP1-AS1 regulates ACVR1, by competitively binding to miR-384 as a competing endogenous RNA (CeRNA).

### Upregulation of miR-384 suppresses PANC-1 cell stemness through down-regulation of ACVR1

**Fig. 4:**
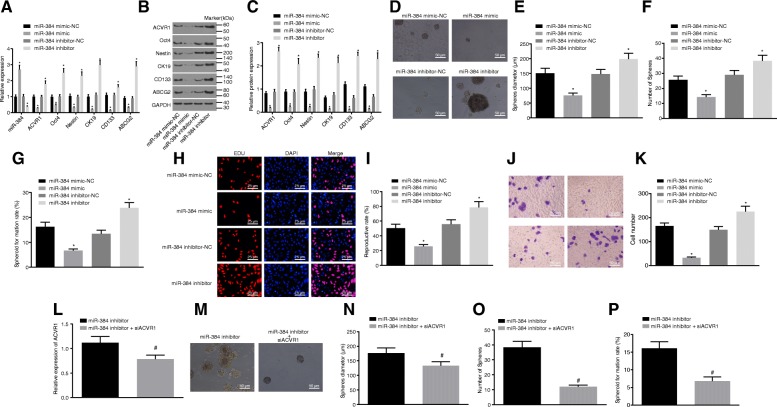
PANC-1 cell stemness is regulated by miR-384 thought targeting ACVR1. **a**, miR-384 expression and mRNA expression of ACVR1 and CSC markers (Oct4, ABCG2, Nestin, CK19 and CD133) as determined by RT-qPCR; **b**, grey value of Oct4, ABCG2, Nestin, CK19, CD133, ACVR1 and GAPDH protein bands in as measured by western blot analysis; **c**, protein expression of Oct4, ABCG2, Nestin, CK19, CD133 and ACVR1; **d**, micrograph of sphere formation of PANC-1 cells (× 200); **e** & **f**, statistical chart of sphere diameter and spheres per 100 cells of PANC-1 cells as determined sphere formation assay; **g**, statistical chart of monoclonal formation rate as evaluated by clone formation assay; **h**, proliferation of PANC-1 cells as assessed by EdU staining (× 200); **i**, statistical chart of cell proliferation; **j**, micrograph of the number of invasive cells as determined by Transwell assay (× 200); **k**, statistical chart of cell invasion; **l**, the mRNA expression of ACVR1 in PANC-1 cells as determined by RT-qPCR; **m**, micrograph of sphere formation of PANC-1 cells (× 200); **n** & **o**, sphere diameter and spheres per 100 cells of PANC-1 cells as determined by sphere formation assay; **p**, monoclonal formation rate as evaluated by clone formation assay; ^*^, *p* < 0.05 vs. the miR-384 mimic-NC and miR-384 inhibitor-NC groups; ^#^, *p* < 0.05 vs. the miR-384 inhibitor group. All the above data were measurement data and expressed as mean ± standard derivation. The *t*-test was performed for comparison between two groups. The experiment was repeated three times. PC, pancreatic cancer; miR-384, microRNA-384; NC, negative control; RT-qPCR, reverse transcription quantitative polymerase chain reaction; GAPDH, glyceraldehyde-3-phosphate dehydrogenase; ACVR1, activin receptor A type I; ABCG2, ATP-binding cassette subfamily G member 2

In order to evaluate the role of miR-384 and ACVR1 in PC, the PANC-1 cell line was transfected with different vectors and further expression of miR-384, ACVR1 and CSC markers was determined by RT-qPCR and western blot analysis (Fig. 4a-c). There was no significant difference detected between the miR-384 mimic-NC and miR-384 inhibitor-NC groups in relation to the expression of miR-384, ACVR1 and CSC markers (Oct4, ABCG2, Nestin, CK19 and CD133) (*p* > 0.05). In contrast to the miR-384 mimic-NC and miR-384 inhibitor-NC groups, the expression of miR-384 increased, ACVR1 decreased as did the CSC markers (Oct4, ABCG2, Nestin, CK19 and CD133) in the miR-384 mimic group (all *p* < 0.05); while a contrasting trend was identified in the miR-384 inhibitor group (*p* < 0.05). Meanwhile, the PANC-1 cell ability after varying transfection was examined. There was no significant difference detected between miR-384 mimic-NC and miR-384 inhibitor-NC groups in terms of sphere number, sphere diameter, monoclonal formation rate, cell proliferation and invasion (*p* > 0.05) (Fig. 4d-k). However, comparing the two NC groups, the miR-384 mimic group exhibited a decreased sphere number, sphere diameter, monoclonal formation rate, cell proliferation and invasion (all *p* < 0.05), all of which were increased in the miR-384 inhibitor group (all *p* < 0.05) (Fig. 4d-k).

Compared with the miR-384 inhibitor group, the expression of ACVR1 was significantly diminished in the miR-384 inhibitor + si-ACVR1 group (*p* < 0.05) (Fig. 4l). Additionally, the sphere number, sphere diameter and monoclonal formation rate was also decreased in the miR-384 inhibitor + si-ACVR1 group (*p* < 0.05) (Fig. 4m-p). Therefore, inhibition of miR-384 enhances PC cell stemness, however, when the expression of ACVR1 was interfered with, the stemness was declined.

### Knockdown of AFAP1-AS1 suppresses tumor formation ability in nude mice by upregulating miR-384

**Fig. 5:**
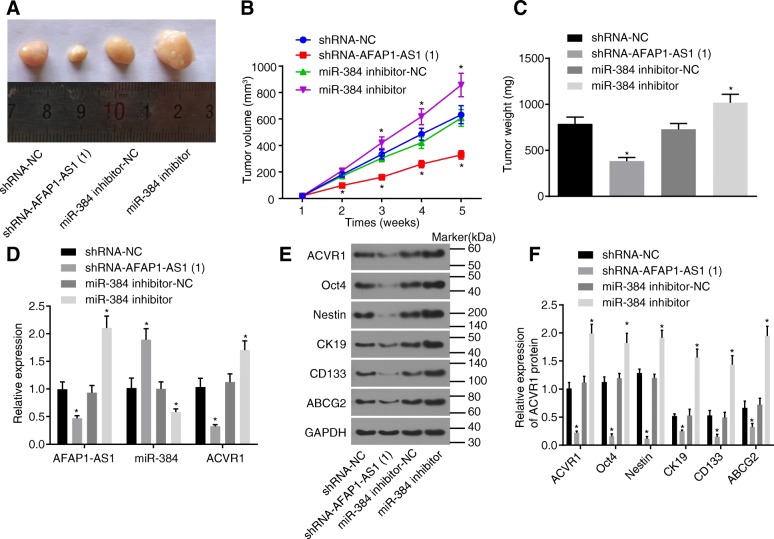
Pancreatic tumorigenesis is restrained by knockdown of AFAP1-AS1 or enhanced by inhibition of miR-384 (*n* = 6). **a**, picture of tumor formation in nude mice transferred with miR-384 inhibitor or shRNA-AFAP1-AS1; **b**, tumor volume of nude mice transferred with miR-384 inhibitor or shRNA-AFAP1-AS1; **c**, tumor weight of nude mice transferred with miR-384 inhibitor or shRNA-AFAP1-AS1; **d**, expression of miR-384, AFAP1-AS1 and ACVR1 in the mice tumor tissues as determined by RT-qPCR; **e** and **f**, the protein expression of ACVR and CSC markers in the mice tumor tissues as measured by western blot analysis; ^*^, *p* < 0.05 vs. the shRNA-NC group. All the above data were measurement data and expressed as mean ± standard derivation. The data of B was analyzed by repeated measurement analysis, and one-way ANOVA was applied for others. The experiment was repeated three times. PC, pancreatic cancer; miR-384, microRNA-384; AFAP1-AS1, actin filament-associated protein 1 antisense RNA 1; NC, negative control; RT-qPCR, reverse transcription quantitative polymerase chain reaction; GAPDH, glyceraldehyde-3-phosphate dehydrogenase; ACVR1, activin receptor A type I; ABCG2, ATP-binding cassette subfamily G member 2

In order to investigate the effect of miR-384 overexpression or knockdown of AFAP1-AS1 on the tumor formation ability, tumor formation in nude mice was conducted. As shown in Fig. 5a-c, compared with the shRNA-NC group, tumor volume and tumor weight of the shRNA-AFAP1-AS1–1 group significantly decreased (*p* < 0.05), while increased in the miR-384 inhibitor group (*p* < 0.05). Next, RT-qPCR and western blot analysis were applied to determine expression of AFAP1-AS1, miR-384, ACVR1 and CSC markers (Oct4, Nestin, CK19, CD133 and ABCG2). As shown in Fig. 5d-f, in comparison with the shRNA-NC group, the expression of AFAP1-AS1, ACVR1, Oct4, Nestin, CK19, CD133 and ABCG2 decreased while miR-384 expression increased in the shRNA-AFAP1-AS1–1 group (*p* < 0.05), while a contrasting tendency was observed in the miR-384 inhibitor group (*p* < 0.05). These results suggested that pancreatic tumorigenesis was repressed by knockdown of AFAP1-AS1, which could be reversed by inhibition of miR-384.

## Discussion

PC represents a troublesome malignancy accompanied with high rates of mortality. A small subpopulation of pancreatic tumor cells with high carcinogenesis called pancreatic CSCs have been shown to lead to the high numbers of PC fatality [19]. LncRNAs have been reported to act as modulators of cellular development and human diseases through the regulation of gene expression [20]. More recently, lncRNAs were implied to exert as a ceRNA for specific miRNAs and regulate their function as well as miRNA target gene expression, affecting cancer development [21, 22]. In the present study, we set out to investigate the underlying mechanism by which the lncRNA AFAP1-AS1 acting as a ceRNA acts to regulate ACVR1 by competitively binding to miR-384, thus playing the role of a carcinogenic factor in the progression of PC.

Initially, bioinformatics prediction suggested that AFAP1-AS1 was up-regulated in PC, both the FISH and RT-qPCR results provided evidence verifying that the up-regulated expression of AFAP1-AS1 in the PC tissues. However, in the PC cells transfected with shRNA-AFAP1-AS1, PC cell sphere formation, proliferation and invasion were all repressed. AFAP1-AS1 has been reported to be up-regulated in a large variety of cancers including that of gallbladder cancer [23] and cholangiocarcinoma [24], whereby the overexpression of AFAP1-AS1 has been implicated in the promotion of cell proliferation. A study previously revealed the effect of AFAP1-AS1 depletion on PDAC, indicating that PDAC cell proliferation, migration and invasion were suppressed [9], all of which was consistent with the findings of our study.

Additionally, the mRNA and protein expression of the CSC markers (Oct4, ABCG2, Nestin, CK19 and CD133) were decreased in the PC cells when AFAP1-AS1 was knocked-down, which ultimately demonstrated that the stemness of the PC cells was attenuated when AFAP1-AS1 was reduced. Stem cell markers possess the ability to control embryonal stem cell self-renewal and differentiation, as well as acting to maintain a variety of biological characteristics [25]. Studies have shown that CSCs isolated form PANC-1 cell line exhibited high expression of CD133/CD44/Oct4/Nestin and resistance to gemcitabine [26]. Oct4 as a marker of CSCs played important role in maintaining CSC stemness, and knockdown of Oct4 inhibited PCSC biologic characteristics, chemoresistance and tumorigenesis, which was demonstrated previously both in vivo and in vitro [27]. Another CSC marker in the form of overexpressed ABCG2 has been linked with the regulation of hypoxia-induced chemoresistance to gemcitabine [28], moreover, ABCG2 expression induced by gastrin has been reported to elevate the proportion of SP cells, increasing the possibility of tumor cell metastasis potential and activity of cell invasion by activating NF-κB signaling in PC [29]. Based on the aforementioned evidence, we subsequently asserted that the self-renewal, stemness of PC cells as well as the biological characteristics of maintenance of CSCs could be suppressed through the knockdown of AFAP1-AS1, which could also lead to the repression of CSC makers.

AFAP1-AS1 was subsequently verified to act as a ceRNA of miR-384 which was confirmed through the application of a dual-luciferase reporter gene assay. AFAP1-AS1/miR-384 coimmunoprecipitation with anti-Ago2 revealed the existence of a physical interaction between themselves among PC cells, further highlighting the activity of AFAP1-AS1 sequestering miRNA. Moreover, the overexpression of miR-384 suppressed PC cell sphere formation, proliferation, invasion and CSC markers expression, which was consistent with the response observed in relation to AFAP1-AS1 knockdown. LncRNAs that act as gene expression regulators by competing for miRNA binding can be defined as ceRNAs [30]. Additionally, down-regulation of miR-384 was revealed in various cancers including non-small-cell lung cancer (NSCLC) [31] and RCC [13], and miR-384 could inhibit NSCLC cell as well as RCC cell growth and invasion through regulation of astrocyte elevated gene-1. In PC, the expression of miR-384 has been previously demonstrated to be inhibited by lncRNA colorectal neoplasia differentially expressed (CRNDE), which on the whole enhanced PC cells proliferation and metastasis by up-regulating insulin receptor substrate 1 (IRS1) through competitively binding to miR-384 [32]. Interestingly, another study also concluded that CRNDE up-regulated pleiotrophin (PTN) by competitively binding to miR-384, while suggesting that the CRNDE/miR-384/PTN axis promoted papillary thyroid cancer cell proliferation, invasion and migration [33]. With the aim of exploring the miR-384 related functions of AFAP1-AS1 in PC pathogenesis, ACVR1 was confirmed to be negatively regulated by miR-384. ACVR1 was further highlighted as a regulator of stem cell markers, with the hepatocellular carcinoma CSC subtype featured by the miR-148a-ACVR1-bone morphogenetic protein (BMP)-Wnt circuit, whereby the expression of ACVR1 was suppressed by miR-148a [14]. Bearing in mind the interaction between AFAP1-AS1 and miR-384, our results obtained further suggested that AFAP1-AS1 could act to regulate the expression of ACVR1 by competitively binding to miR-384, which signifies the role of AFAP1-AS1 in the regulatory network involving PC tumorigenesis.

## Conclusions

**Fig. 6:**
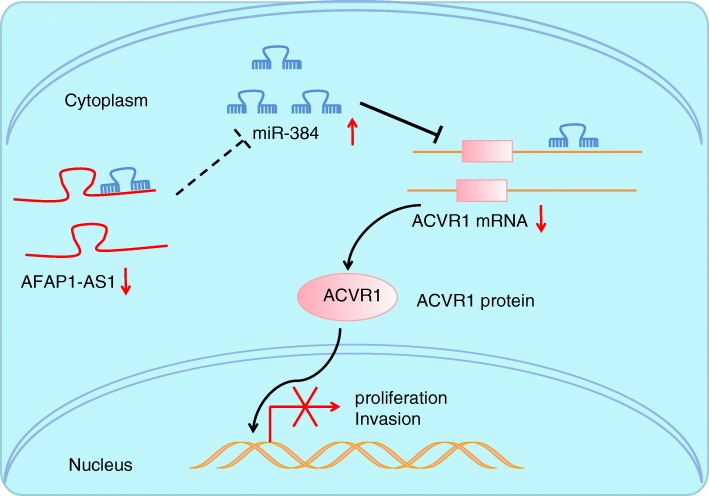
The mechanism of AFAP1-AS1 regulating PC cell stemness. High expression of AFAP1-AS1 was found in PC cells, while down-regulation of AFAP1-AS1 enhanced the expression of miR-384. While up-regulation of miR-384 inhibits the expression of ACVR1, which suppresses PC cell proliferation and invasion. AFAP1-AS1, actin filament-associated protein 1 antisense RNA 1; ACVR1, activin receptor A type I; PC, pancreatic cancer; miR-384, microRNA-384

Taken together, the key observations of our study suggested that the knockdown of AFAP1-AS1 could down-regulate the expression of ACVR1 through the attenuated ability of competitively binding to miR-384, by this way, PC cell proliferation, invasion, migration, stemness and which ultimately acts to restrain tumorigenicity (Fig. 6). The results of the current study provide evidence highlighting a positive AFAP1-AS1/ACVR1 correlation and the cross talk among miR-384, AFAP1-AS1 and ACVR1, and providing a new dimension for PC treatment. However, further studies are still required to investigate the effectiveness and safety of therapeutic potential of the AFAP1-AS1/miR-384/ACVR1 pathway in PC.

## Additional files

Additional file 1: **Figure S1.** AFAP1-AS1 is highly expressed in CSCs derived from SW1990 and Capan-1 cells. A and C, the SP cells with CSC-like properties derived from SW1990 and Capan-1 cell lines sorted by flow cytometry; B and D, RT-qPCR analysis of the expression of CSC markers Oct4, ABCG2, Nestin, CK19 and CD133 as well as AFAP1-AS1 and ACVR1 in SP cells and non-SP cells derived from SW1990 and Capan-1 cell lines. ^, *p* < 0.05 vs. the SP cells. All the above data were measurement data and expressed as mean ± standard derivation, and analyzed by *t*-test. The experiment was repeated 3 times. PC, pancreatic cancer; miR-384, microRNA-384; AFAP1-AS1, actin filament-associated protein 1 antisense RNA 1; NC, negative control; RT-qPCR, reverse transcription quantitative polymerase chain reaction; GAPDH, glyceraldehyde-3-phosphate dehydrogenase; ACVR1, activin receptor A type I; ABCG2, ATP-binding cassette subfamily G member 2; SP, side population. (EPS 2443 kb)

Additional file 2: **Figure S2** AFAP1-AS1 maintains SW1990 cell stemness. A, RT-qPCR analysis of AFAP1-AS1 expression; B and C, western blot analysis of expression of CSC markers; D, sphere formation of SW1990 cells (× 200); E and F, sphere diameter and the number of spheres per 100 cells of SW1990 cells determined by sphere formation assay; G, monoclonal formation rate evaluated by colony formation assay; ^*^, *p* < 0.05 vs. the shRNA-NC group. #, *p* < 0.05 vs. the empty vector group. All the above data was measurement data and expressed as mean ± standard derivation. One-way ANOVA was applied for comparison among three groups. The *t*-test was performed for comparison between two groups. The experiment was repeated three times. AFAP1-AS1, actin filament-associated protein 1 antisense RNA 1; PC, pancreatic cancer; RT-qPCR, reverse transcription quantitative polymerase chain reaction; GAPDH, glyceraldehyde-3-phosphate dehydrogenase; ACVR1, activin receptor A type I; ABCG2, ATP-binding cassette subfamily G member 2. (EPS 8547 kb)

## Abbreviations

ABCG2: breast cancer resistance protein
ACVR1: Activin receptor A type I
AFAP1-AS1: Actin filament-associated protein 1 antisense RNA 1
AGO2: Argonaute 2
ANOVA: Analysis of variance
BCA: Bicinchoninic acid
BCIP: Bromo-4-chloro-3-indolyl phosphate
BSA: Bovine serum albumin
CeRNA: Competing endogenous RNA
CK19: keratin 19
CSCs: Cancer stem cells
DAPI: Diamidino-2-phenylindole
DMEM: Dulbecco’s modified Eagles Medium
dNTP: deoxy-ribonucleoside triphosphate
ECL: Electrogenerated chemiluminescence
EDTA: ethylene diamine tetraacetic acid
EdU: 5-ethynyl-2′-deoxyuridine
EP: Eppendorf
FBS: Fetal bovine serum
FISH: Fluorescence in situ hybridization
GAPDH: Glyceraldehyde-3-phosphate dehydrogenase
IgG: Immunoglobulin G
lncRNAs: long non-coding RNAs
MALAT-1: Metastasis-associated lung adenocarcinoma transcript 1
miR: microRNA
miR-384: microRNA-384
MMLV: Moloney Murine Leukemia Virus
Mut: Mutant
NBT: Nitro blue tetrazolium
NC: Negative control
Oct4: Organic cation/carnitine transporter 4
PBS: Phosphate buffer saline
PBST: Poly (butylene succinate-co-terephthalate)
PC: Pancreatic cancer
PC: Pancreatic cancer
PDAC: Pancreatic ductal adenocarcinoma
PMFS: Phenylmethylsulfonyl fluoride
PVDF: Polyvinylidene fluoride film
RCC: Renal cell cancer
RIPA: Radioimmunoprecipitation
RIPA: Radioimmunoprecipitation assay
RT-qPCR: Reverse transcription quantitative polymerase chain reaction
SP: Side population
SPF: Specific-pathogen-free
TBST: Tris-buffered saline with Tween 20
TNM: Tumor-node-metastasis
Wt: Wide type

## References

[1] Zhao L, Kong H, Sun H, Chen Z, Chen B, Zhou M (2018). LncRNA-PVT1 promotes pancreatic cancer cells proliferation and migration through acting as a molecular sponge to regulate miR-448. J Cell Physiol.

[2] Zhang SH, Liu GF, Li XF, Liu L, Yu SN (2018). Efficacy of different chemotherapy regimens in treatment of advanced or metastatic pancreatic cancer: a network meta-analysis. J Cell Physiol.

[3] Zhou Y, Shan T, Ding W, Hua Z, Shen Y, Lu Z (2018). Study on mechanism about long noncoding RNA MALAT1 affecting pancreatic cancer by regulating hippo-YAP signaling. J Cell Physiol.

[4] Yang Z, Tang Y, Lu H, Shi B, Ye Y, Xu G (2018). Long non-coding RNA reprogramming (lncRNA-ROR) regulates cell apoptosis and autophagy in chondrocytes. J Cell Biochem.

[5] Bo H, Gong Z, Zhang W, Li X, Zeng Y, Liao Q (2015). Upregulated long non-coding RNA AFAP1-AS1 expression is associated with progression and poor prognosis of nasopharyngeal carcinoma. Oncotarget.

[6] Wang F, Ni H, Sun F, Li M, Chen L (2016). Overexpression of lncRNA AFAP1-AS1 correlates with poor prognosis and promotes tumorigenesis in colorectal cancer. Biomed Pharmacother.

[7] Shi X, Zhang H, Wang M, Xu X, Zhao Y, He R (2017). LncRNA AFAP1-AS1 promotes growth and metastasis of cholangiocarcinoma cells. Oncotarget.

[8] Liu FT, Xue QZ, Zhu PQ, Luo HL, Zhang Y, Hao T (2016). Long noncoding RNA AFAP1-AS1, a potential novel biomarker to predict the clinical outcome of cancer patients: a meta-analysis. Onco Targets Ther.

[9] Ye Y, Chen J, Zhou Y, Fu Z, Zhou Q, Wang Y (2015). High expression of AFAP1-AS1 is associated with poor survival and short-term recurrence in pancreatic ductal adenocarcinoma. J Transl Med.

[10] Ogawa T, Hirohashi Y, Murai A, Nishidate T, Okita K, Wang L (2017). ST6GALNAC1 plays important roles in enhancing cancer stem phenotypes of colorectal cancer via the Akt pathway. Oncotarget.

[11] Jiao F, Hu H, Han T, Yuan C, Wang L, Jin Z (2015). Long noncoding RNA MALAT-1 enhances stem cell-like phenotypes in pancreatic cancer cells. Int J Mol Sci.

[12] Wang L, Bu P, Ai Y, Srinivasan T, Chen HJ, Xiang K, et al. A long non-coding RNA targets microRNA miR-34a to regulate colon cancer stem cell asymmetric division. elife. 2016;5. 10.7554/eLife.14620 PMC4859802 27077950

[13] Song H, Rao Y, Zhang G, Kong X (2017). MicroRNA-384 inhibits the growth and invasion of renal cell carcinoma cells by targeting astrocyte elevated gene 1. Oncol Res.

[14] Li L, Liu Y, Guo Y, Liu B, Zhao Y, Li P (2015). Regulatory MiR-148a-ACVR1/BMP circuit defines a cancer stem cell-like aggressive subtype of hepatocellular carcinoma. Hepatology.

[15] Wang Y, Zhang Z, Wang J (2018). MicroRNA-384 inhibits the progression of breast cancer by targeting ACVR1. Oncol Rep.

[16] Dassanayake DL, Muthunayake TM, Senevirathna KH, Siribaddana A (2012). Staging of lung cancer in a tertiary care setting in Sri Lanka, using TNM 7th edition. A comparison against TNM6. BMC Res Notes.

[17] Luo L, Yang R, Zhao S, Chen Y, Hong S, Wang K (2018). Decreased miR-320 expression is associated with breast cancer progression, cell migration, and invasiveness via targeting aquaporin 1. Acta Biochim Biophys Sin Shanghai.

[18] Livak KJ, Schmittgen TD (2001). Analysis of relative gene expression data using real-time quantitative PCR and the 2(−Delta Delta C(T)) method. Methods.

[19] Yang Z, Zhang Y, Tang T, Zhu Q, Shi W, Yin X (2018). Transcriptome profiling of Panc-1 spheroid cells with pancreatic Cancer stem cells properties cultured by a novel 3D semi-solid system. Cell Physiol Biochem.

[20] Zhao L, Sun H, Kong H, Chen Z, Chen B, Zhou M (2017). The Lncrna-TUG1/EZH2 Axis promotes pancreatic Cancer cell proliferation, migration and EMT phenotype formation through sponging Mir-382. Cell Physiol Biochem.

[21] Liu XH, Sun M, Nie FQ, Ge YB, Zhang EB, Yin DD (2014). Lnc RNA HOTAIR functions as a competing endogenous RNA to regulate HER2 expression by sponging miR-331-3p in gastric cancer. Mol Cancer.

[22] Zhao L, Han T, Li Y, Sun J, Zhang S, Liu Y (2017). The lncRNA SNHG5/miR-32 axis regulates gastric cancer cell proliferation and migration by targeting KLF4. FASEB J.

[23] Ma F, Wang SH, Cai Q, Zhang MD, Yang Y, Ding J (2016). Overexpression of LncRNA AFAP1-AS1 predicts poor prognosis and promotes cells proliferation and invasion in gallbladder cancer. Biomed Pharmacother.

[24] Lu X, Zhou C, Li R, Deng Y, Zhao L, Zhai W (2017). Long noncoding RNA AFAP1-AS1 promoted tumor growth and invasion in cholangiocarcinoma. Cell Physiol Biochem.

[25] Freitag D, McLean AL, Simon M, Koch A, Grube S, Walter J (2017). NANOG overexpression and its correlation with stem cell and differentiation markers in meningiomas of different WHO grades. Mol Carcinog.

[26] Wang D, Zhu H, Zhu Y, Liu Y, Shen H, Yin R (2013). CD133(+)/CD44(+)/Oct4(+)/nestin(+) stem-like cells isolated from Panc-1 cell line may contribute to multi-resistance and metastasis of pancreatic cancer. Acta Histochem.

[27] Lu Y, Zhu H, Shan H, Lu J, Chang X, Li X (2013). Knockdown of Oct4 and Nanog expression inhibits the stemness of pancreatic cancer cells. Cancer Lett.

[28] He X, Wang J, Wei W, Shi M, Xin B, Zhang T (2016). Hypoxia regulates ABCG2 activity through the activivation of ERK1/2/HIF-1alpha and contributes to chemoresistance in pancreatic cancer cells. Cancer Biol Ther.

[29] Wang J, Xin B, Wang H, He X, Wei W, Zhang T (2016). Gastrin regulates ABCG2 to promote the migration, invasion and side populations in pancreatic cancer cells via activation of NF-kappaB signaling. Exp Cell Res.

[30] Fu Z, Li G, Li Z, Wang Y, Zhao Y, Zheng S (2017). Endogenous miRNA sponge LincRNA-ROR promotes proliferation, invasion and stem cell-like phenotype of pancreatic cancer cells. Cell Death Discov.

[31] Fan N, Zhang J, Cheng C, Zhang X, Feng J, Kong R (2017). MicroRNA-384 represses the growth and invasion of non-small-cell lung cancer by targeting astrocyte elevated gene-1/Wnt signaling. Biomed Pharmacother.

[32] Wang G, Pan J, Zhang L, Wei Y, Wang C. Long non-coding RNA CRNDE sponges miR-384 to promote proliferation and metastasis of pancreatic cancer cells through upregulating IRS1. Cell Prolif. 2017;50. 10.1111/cpr.12389 PMC6529119 28940804

[33] Sun H, He L, Ma L, Lu T, Wei J, Xie K (2017). LncRNA CRNDE promotes cell proliferation, invasion and migration by competitively binding miR-384 in papillary thyroid cancer. Oncotarget.

